# Preparation of Novel Slow-Release Acid Materials for Oilfield Development via Encapsulation

**DOI:** 10.3390/ma18010083

**Published:** 2024-12-28

**Authors:** Xinshu Sun, Chen Chen, Mingxuan Li, Yiming Yao, Baohua Guo, Jun Xu

**Affiliations:** 1 Department of Chemical Engineering, Tsinghua University, Beijing 100084, China; sxs23@mails.tsinghua.edu.cn (X.S.); bhguo@tsinghua.edu.cn (B.G.); 2 Sinopec Research Institute of Petroleum Engineering Co., Ltd., Beijing 102206, China; chenchen.sripe@sinopec.com (C.C.); limx0613.sripe@sinopec.com (M.L.); yaoym.sripe@sinopec.com (Y.Y.)

**Keywords:** oil and gas extraction engineering, acid-fracturing technology, slow-release acid generation, microcapsules

## Abstract

Acid-fracturing technology has been applied to form pathways between deep oil/gas resources and oil production pipelines. The acid fracturing fluid is required to have special slow-release performance, with no acidity at low temperatures, while steadily generating acid at high temperatures underground. At present, commercial acid systems in oilfields present problems such as the uncontrollable release effect, high costs, and significant pollution. In this research, we designed an innovative chloroformate material and investigated the release of the acid at various temperatures. This new chloroformate material reacts slowly with water at room temperature, and can completely react with water to form hydrochloric acid at high temperatures, without residual organic chlorine and other harmful substances; thus, it is suitable for use as an acid agent in oilfields. To isolate the acid-release core material from the outer water phase, we encapsulated the former with various materials, such as cross-linked polyacrylate or polystyrene, to obtain microcapsules. By slowly breaking and degrading the shell layer at a high temperature, the goal of no acid being released at low temperatures with slow acid generation at a high temperature was achieved. The microcapsules were prepared using radical polymerization and the phase separation method. Furthermore, scanning electron microscopy, differential scanning calorimetry, chemical titration analysis, and other methods were used to characterize the structure and the sustained acid release of microcapsules. The results of thermogravimetry and other experiments showed that the prepared microcapsules successfully coated the chloroformate material. In contrast to the bare material, the slow-release performance of the microcapsules was significantly improved, and the continuous acid generating time was able to reach more than 10 h. Under optimum conditions, microcapsules with a uniform particle size with a sustained-release acid core were prepared, and the encapsulation efficiency reached up to 60%. Compared with traditional acid-release systems, the new system prepared in this study has better acid-release performance at high temperatures, while the product is both clean and convenient to use. Multiple important parameters, such as microcapsule particle size, can also be controlled by varying the experimental conditions to meet the needs of different oil/gas extraction environments. In summary, we prepared a promising new and efficient slow-release acid generation system, which has unique practical significance for optimizing current oilfield acid-fracturing technology.

## 1. Introduction

In recent years, with the acceleration of global industrialization and urbanization, the demand for energy, particularly oil and gas resources, has been rapidly increasing. As a result, current energy exploration objectives are gradually shifting from conventional shallow-layer oil and gas resources to high-temperature deep-layer unconventional oil and gas resources, such as tight gas, coalbed gas, and shale oil. Deep-layer oil and gas exploration processes differ significantly from ordinary processes and require the use of multiple new technologies to efficiently extract oil and gas resources under the harsh conditions of high temperature and high pressure. Among them, acid fracturing technology, as a key technology for rock modification, has played a huge role in the development of unconventional oil and gas resources [1,2].

High-temperature, deep-layer, unconventional hydrocarbon resources are mainly stored in carbonate rocks, which have poor homogeneity, complex geological structures, and are difficult to access using conventional vertical drilling technology. Additionally, conventional physical exploration technologies cannot accurately map the geological structure, making oil and gas exploration in deep, complex structures challenging. Currently, the development and utilization of this part of hydrocarbon resources rely heavily on acid fracturing technology. In this process, the acid solution serves as a pressure transmission source in the first stage to break the rock layers and form multiple deep cracks to artificially construct the passage between the deep hydrocarbons and the main passage. In the second stage, the acid solution acts as a corrosive agent to further destroy the carbonate rock layers, expanding the hydrocarbon passage while clearing the carbonate rock debris and powder formed by the fracturing, avoiding clogging of the hydrocarbon passage, and thus improving extraction efficiency [3,4,5,6].

Compared with earlier sand-fracturing technology used in oilfield exploitation, acid-fracturing technology only needs to inject acid solution into the pipeline, which is more convenient for pipeline transportation as it does not require external proppants to maintain the crack shape. In addition, the acid solution flows and corrodes more flexibly in the rock fissures, blocking the oil and gas channels to a lesser extent, resulting in higher permeability and reservoir transformation efficiency. Due to their high porosity, abundant natural fractures, and the brittleness of carbonate rocks storing unconventional oil and gas, it is difficult to add proppants, and the acid-fracturing technology has a unique advantage for carbonate reservoirs. Therefore, the acid-fracturing technology is gradually replacing traditional sand-fracturing technology and has become one of the most widely used and studied technologies for the exploitation of unconventional oil and gas resources [7,8].

For acid-fracturing technology, the key to research lies in selecting appropriate fracturing acid solutions. During the acid-fracturing process, the acid solution needs to act as a fracturing medium in the first stage and perform rock corrosion in the second stage. If only ordinary acid solutions, such as hydrochloric acid, are used for acid-fracturing operations, they will rapidly corrode the rock layers in contact with them under high-temperature and high-pressure conditions of the reservoir (pressure of about 20–40 MPa and temperature above 150 °C), which greatly shortens the effective action distance of acidizing and thus cannot construct an effective passage between the main exploitation pipeline and deep oil and gas reservoirs, reducing the exploitation efficiency [9]. Therefore, the ideal performance of the acid solution is to weaken the corrosion to the rock layers as much as possible in the fracturing stage and then perform the rock corrosion modification after the stage is over. Currently, the acid-fracturing technology mainly replaces ordinary acid solutions with special formulations of slow-releasing acid systems that do not show acidity at room temperature and slowly generate acidizing corrosion capacity under high-temperature and high-pressure conditions [10]. Developing slow-releasing acid formulations with better performance to meet the requirements of the deep-layer oil and gas exploitation industry has become a crucial area of research for acid fracturing technology.

**Figure 1:**
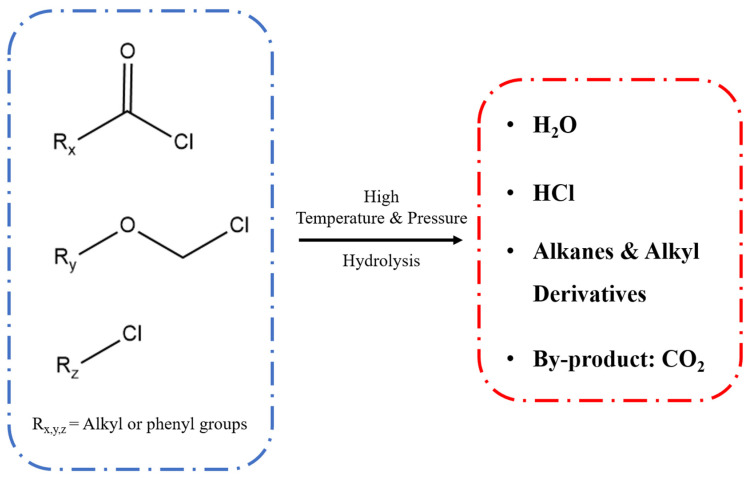
The composition, hydrolysis process, and generated products of the innovative acid-generating compounds.

In conventional acid release systems, hydrochloric acid or similar simple inorganic acids are commonly used as the acid source. Due to the strong acidity of hydrochloric acid under all conditions and it being a volatile liquid that is difficult to store and transport, it performs poorly as a self-generating acid material in acid release systems, especially in encapsulated acid systems where hydrochloric acid solutions are difficult to encapsulate, increasing the difficulty of industrial production [11]. Therefore, it is necessary to choose suitable acid-generating agents to replace the commonly used hydrochloric acid solution. At present, the acid-generating agents used in acid release systems mainly include organic acid esters, a mixture of ammonium chloride and formaldehyde, halogenated hydrocarbons, acyl chlorides, etc. All of them have obvious drawbacks, such as high cost, insufficient acidity of the generated organic acids, being difficult to control acid generation, and large environmental pollution of the ammonium chloride and formaldehyde mixture [12,13]. In recent years, a variety of new slow-release acid generation systems have been developed to overcome the shortcomings of traditional acid generation agents. For example, asphalt, diesel, and ordinary acid were mixed to delay the reaction time of hydrogen ions through the oil phase [14]. Quaternary ammonium salt and other additives were used to increase the acid stability and acid etching temperature [15]. Urea derivatives were used as new slow-release materials instead of traditional acidifiers [16], etc. The above studies were able to partially improve the acidizing effect of the etchant in the formulation, but the delayed acid generation capacity and continuous acid generation ability were still unable to fully meet the growing industrial demand. At the same time, the emulsification system and unstable slow-release materials pose challenges to industrial production, storage, and transportation. To solve these problems, this study screened a kind of chloro-contained mixture as acid-generating material. This mixture was invented by the Sinopec Research Institute of Petroleum Engineering Co., Ltd. and was intended to be used for commercial purposes. The specific components are mixtures of chloroformate, combined with various alkyl or aryl acyl chlorides, chlorinated hydrocarbons, and chloromethyl ethers. Through relatively fine component adjustment, this acid-generating material with high chlorine content is relatively stable at room temperature and reacts slowly with water, as shown in the test results below. A protective layer, composed of polystyrene, polymethylmethacrylate, or other common polymers, was formed on the surface of the acid-generating material by encapsulation to further slow its acid release rate, thus preparing a series of new acid release systems. When the suspension formed by mixing this system with water is injected into the stratum, under the actions of high temperature and high pressure, the outer polymer layer will slowly hydrolyze and age, releasing the inner acid-generating material after a long time, and then hydrolyze to release hydrochloric acid, water, and hydrocarbon derivatives, as well as a byproduct of carbon dioxide, as shown in Figure 1. The hydrocarbon derivatives produced in the whole process are similar to those contained in the extracted oil, which will not damage the quality of the oil. Except for acid corrosion, carbon dioxide and hydrochloric acid will not have any impact on the environment at the same time. Therefore, this system is environmentally friendly when used and will not introduce new harmful residues to the ground.

## 2. Methods

The experimental reagents used in this study were as follows: chloroformate, azo-bisisobutyronitrile (AIBN), sodium silicate, and dodecyl trimethylbenzene sulfonate cationic surfactant (CTAOTs) were purchased from Shanghai Aladdin Biochemical Technology Co., Ltd. (Shanghai, China). Methyl methacrylate (MMA), xylene, octylphenol polyoxyethylene ether (OP-10), and trimethylolpropane triacrylate (TMPTA) were purchased from Beijing Honghu Joint Chemical Co., Ltd. (Beijing, China). Dimethyl carbonate (DMC) and paraffin were purchased from Beijing Tongguang Fine Chemical Co., Ltd. (Beijing, China). All monomers for polymerization were treated to remove polymerization inhibitors before use, and active initiators such as azobisisobutyronitrile were purified by recrystallization before use.

### 2.1. Encapsulation of Pure Oil

Before encapsulating the acid-release solid powders, we used pure oil phase as the core material for the encapsulation study to determine the optimal encapsulation method. Three different encapsulation methods were investigated: encapsulation via the radical polymerization of acrylate, via silica precipitation, and via phase separation.

The representative encapsulation method via radical polymerization is as follows: 0.3 g of ammonium persulfate, as a water-phase initiator, was added to 200 mL of a 2 wt% polyvinyl alcohol aqueous solution. Furthermore, 7.2 g of methyl methacrylate and 0.8 g of trimethylolpropane triacrylate (both treated with an alumina column to remove inhibitors) were mixed with 16 g of oil phase liquid and stirred at 200 rpm for 10 min with a magnetic stirrer to ensure thorough mixing. Under stirring at 2000 rpm, the well-mixed oil phase was added to the aqueous solution with the temperature maintained at 35 °C for continuous stirring for 10 min. The temperature was then rapidly raised to 70 °C and maintained for 2 h, followed by raising the temperature to 85 °C and keeping it for 1 h to further solidify the microcapsules. After the reaction system was cooled to room temperature, the microcapsules were filtered and washed alternately with water and ethanol. The microcapsules obtained were dried at 60 °C under vacuum for 24 h. In the case of using an oil-phase initiator, 0.3 g of azobisisobutyronitrile was dissolved in 3 g of dimethyl carbonate. Then, 7.2 g of methyl methacrylate and 0.8 g of trimethylolpropane triacrylate (both treated with an alumina column to remove inhibitors) were taken, and the above species were mixed with 13 g of oil phase liquid (except for the oil phase components of the polymer monomer and crosslinking agent, which were fixed at 16 g). The mixture was stirred at 200 rpm for 10 min with a magnetic stirrer to ensure thorough mixing. Under stirring at 2000 rpm, the well-mixed oil phase was added to pre-configured 200 mL of a 2 wt% polyvinyl alcohol aqueous solution, and the temperature was maintained at 35 °C for continuous stirring for 10 min. The temperature was then rapidly raised to 70 °C and maintained for 2 h, followed by raising the temperature to 85 °C to further solidify the microcapsules for 1 h. After the reaction was completed, the system was cooled to room temperature, filtered, and washed alternately with water and ethanol. The microcapsules obtained were dried at 60 °C under vacuum for 24 h. Parameters, such as the stirring speed, the oil phase type, and the oil phase proportion, were modified in the experiment to explore the optimal encapsulation scheme.

The representative encapsulation method via silica precipitation involves accurately weighing 1 g of nonionic surfactant OP-10 and 1.5 g of cationic surfactant CTAOTs, which were added to a 100 mL aqueous TsOH solution. The mixture was stirred at 300 rpm for 10 min, followed by the addition of 20 g of oil phase liquid under stirring at 300 rpm for 30 min at room temperature to achieve uniform emulsification. The temperature was then raised to 45 °C, and 100 mL of 0.4 M sodium silicate aqueous solution was slowly added dropwise. The reaction was allowed to solidify for 2 h. The resulting product was washed alternately with water and cyclohexane and filtered. The microcapsules obtained were dried at 60 °C under vacuum for 24 h.

The following is a representative phase separation coating method: A precise amount of 0.3 g of cationic surfactant CTAOTs was accurately weighed and added to 200 mL of water, and the mixture was stirred at 200 rpm until it was homogeneous. Subsequently, 1.5 g of polystyrene and 5 g of chloroformate were added to 40 g of dichloromethane at room temperature and stirred at 200 rpm for 30 min to ensure the complete dissolution of the solid. This solution was then added dropwise to the aqueous phase and stirred at 200 rpm while heating to 40 °C and maintaining the temperature for 6 h. Finally, the solid was obtained by filtration.

Finally, a controlled-release acid system was prepared by replacing petroleum with a chloroformate solution based on the above series of experiments. After the preparation of the controlled-release acid system, hydrolysis experiments, nuclear magnetic resonance (NMR), and dynamic thermal mechanical analysis (DTA) were conducted to characterize the ratio of chloroformate encapsulation and release performance.

Then, 1 g of dried controlled-release acid was accurately weighed and placed in the inner chamber of a 50 mL hydrothermal reactor, and 10 mL of distilled water was accurately measured using a pipette and added to the inner chamber. After the installation of the hydrothermal reactor was completed, it was placed in an explosion-proof oven and heated at 180 °C for 24 h before being removed. After adequate cooling, 1 mL of residual liquid was taken, diluted with 9 mL of distilled water, and the pH value was measured as q. The chloroformate coating rate T was calculated as follows:

(1) $$T=\frac{10^{-1-q}}{6}\times 276.1\times 100\%$$

An additional set of 1 g dry controlled-release acids was placed in the inner chamber of a 50 mL hydrothermal reactor, with 10 mL distilled water accurately measured and added to the inner chamber using a micropipette. After assembly of the hydrothermal reactor, it was heated at different temperatures (60–160 °C) in an explosion-proof oven for 6 h, then removed and allowed to cool thoroughly. Subsequently, 1 mL of the residual solution was taken, diluted with 9 mL of distilled water, and the pH value was measured. These pH values were combined with the previously measured chloroformate encapsulation rates to determine the hydrolysis rates of each group, which were compared with the hydrolysis data of chloroformate in the bulk at different temperatures.

### 2.2. Determination of Hydrolysis Rate and Properties of Chloroformate Under Different Conditions

An accurately weighed 0.297 g (0.001 mol) acid-release solid mixture, chloroformate, was placed in a 50 mL thick-walled pressure-resistant bottle, and 6 mL of distilled water was accurately added using a pipette, labeled as the solid acid-release group. Three 1 M solutions of chloroformate in dimethylbenzene were placed in three 50 mL thick-walled pressure-resistant bottles, and 6 mL of distilled water was accurately added to each using a pipette and labeled as solution acid-release groups 1, 2, and 3. The four pressure-resistant bottles were placed in a room temperature environment for hydrolysis, and groups 2 and 3 of the solution acid-release system were respectively subjected to 300 r/min and 600 r/min stirring. Every 5 h, 0.1 mL of solution was taken from each group, diluted to 10 mL, and the pH was measured. The acidity of the solution can be calculated from the measured pH value, and the hydrolysis rate of each group of acid-release material can be calculated since the acidity is entirely provided by the hydrolysis of acid-release material to form hydrochloric acid. The experiment was repeated at 70 °C to investigate the effects of phase state (solid and solution) and stirring on the hydrolysis of acid-release material at room temperature and high temperature. Repeating the experiment by altering conditions such as temperature, pH, and so forth, following the aforementioned procedure, will yield kinetic data for the hydrolysis of chloroformate under various conditions.

### 2.3. Determination of Organic Chlorine Content in Hydrolysis Products

In this study, the organic chloride in the hydrolysis residue was converted to inorganic chloride for determination using chemical oxidation. 0.3944 g of thiocyanic acid was accurately weighed, dissolved in water, and diluted to 250 mL to obtain a standard solution of thiocyanic acid, and its accurate concentration was determined by titration with 0.1 mol/L silver nitrate solution. Then, 1 mL of the sample solution was dissolved in 50 mL of water; 5 mL of 6 M nitric acid and 1 mL of 0.0015 M ferrous ammonium sulfate indicator were added, followed by 1 mL of nitrobenzene. Then, 10 mL of 0.1 mol/L silver nitrate solution was added, and the solution was thoroughly mixed. The thiocyanic acid standard solution was titrated until the solution turned deep red, and the concentration of the thiocyanic acid standard solution was recorded as *c*_1_ (in mol/L), and the volume of the thiocyanic acid standard solution consumed was recorded as v (in mL). The concentration of chloride ions *c* (in mg/mL) in the sample solution can be calculated using the following formula:

(2) $$c=35.45\times \left(1-vc_{1}\right)$$

By using this method, the initial inorganic chloride ion concentration in the sample solution can be measured. Another 1 mL of the sample solution is added dropwise to the mixed solution of 3 mL concentrated H_2_SO_4_ + 1 mL 30% H_2_O_2_, and the resulting solution is vigorously stirred. Then, 5.52 mL of 10 mol/L Ba(NO_3_)_2_ is added, and the titration is carried out using the aforementioned method to measure the total chloride concentration (expressed as chloride ions) in the sample solution. The organic chloride concentration in the sample solution is obtained by subtracting the inorganic chloride ion concentration from the total chloride concentration.

## 3. Results and Discussions

### 3.1. Analysis of the Hydrolysis Performance of Chloroformate in Different States

**Figure 2:**
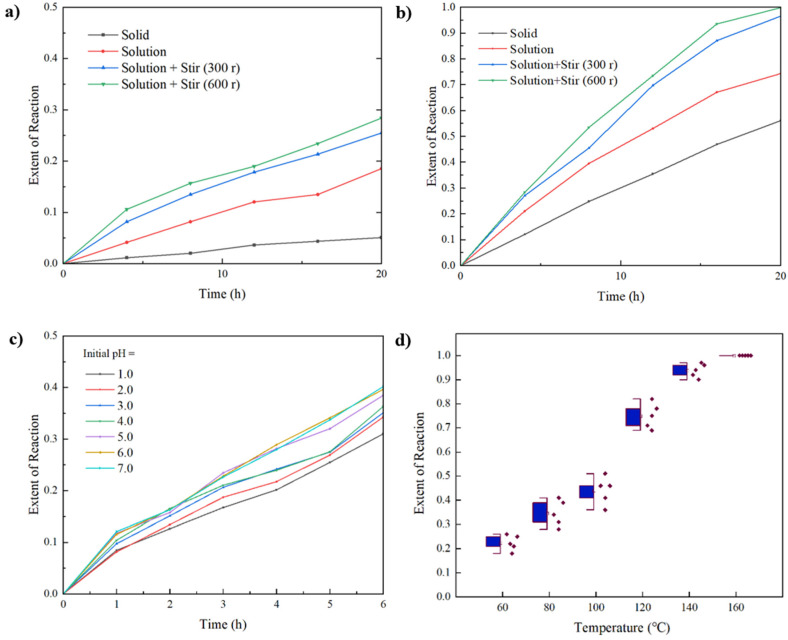
Hydrolysis degree of different forms of the chloroformate (**a**) at room temperature, (**b**) at 70 °C over time. (**c**) Hydrolysis degree at 70 °C with different initial pH levels over time. (**d**) Extent of reaction of the chloroformate after 6-h hydrolysis at different temperatures (each temperature measured five times).

In the experiment, the hydrolysis of chloroformate was measured under four different conditions: at room temperature, at an elevated temperature (70 °C), dissolved in a xylene solution (3 mol/L), dissolved in a xylene solution with slow stirring (300 rpm), and dissolved in a xylene solution with fast stirring (600 rpm). The experimental results are shown in Figure 2a,b. From the experimental results, it is evident that the hydrolysis rate of the oil-phase solution of chloroformate after mixing with water is significantly faster than that of the solid-phase acid-release material reacting with water. It is speculated that the contact surface area between the oil-phase solution and water is much larger than that of large solid particles, thus facilitating hydrolysis. This phenomenon is more pronounced at higher temperatures. Additionally, a higher stirring speed further increased the hydrolysis reaction rate, presumably because high-speed stirring broke the oil-phase solution into finer droplets, further increasing the interfacial contact area and enhancing the hydrolysis rate. At room temperature, the reaction rate of chloroformate with water is not rapid. This is attributed to kinetic factors: the chloroformate is insoluble in the aqueous phase, thereby hindering its reaction with water at normal temperatures. Consequently, the chloroformate is relatively safe at room temperature, as it does not decompose due to moisture over long-term storage. This negates the need for storing components A and B separately, as required by traditional slow-release acid agents, highlighting one of the advantages of using chloro-carbonate as a slow-release acid agent. At elevated temperatures, the hydrolysis rates of chloro-carbonate in all four states were significantly faster, with reactions being more vigorous under high-speed stirring. It is hypothesized that at high temperatures, increased molecular movement and collisions overcome the kinetic barriers to hydrolysis. The hydrolysis results of the chloroformate in different states support the theoretical premise that it possesses slow-release hydrolysis properties.

Due to the possibility that different encapsulation methods may result in varying acidity conditions, which can influence the hydrolysis rate of the chloroformate to some extent, the experiment substituted water with sulfuric acid solutions of different pH values to study the hydrolysis reaction of the chloroformate. This was done to explore the effect of different initial pH levels on the hydrolysis at a fixed temperature of 70 °C. The experimental results are shown in Figure 2c. An environment with higher initial acidity can somewhat slow down the reaction, with the effect becoming more pronounced as the pH decreases. A theoretical analysis of this phenomenon suggests that a stronger acidic environment can lead to more protonation of the species attacking the chloroformate, thereby weakening their nucleophilic capability. Therefore, the encapsulation of the chloroformate should be done in a strong acidic environment or using methods that can withstand acidic conditions.

To determine the temperature required for the hydrolysis of the chloroformate, this study selected a temperature range from 60 °C to 160 °C, with measurements taken at 20 °C intervals. Each temperature was measured five times to obtain a correlation graph between the hydrolysis degree and temperature. The measurement results are shown in Figure 2d. As shown in the figure, the hydrolysis degree at lower temperatures (below 100 °C) ranges between 30% and 50% after 6 h, with the hydrolysis degree increasing as the temperature rises, indicating a certain slow-release performance. When the temperature exceeds 100 °C, the water reaches its boiling point under atmospheric pressure in a sealed environment, causing the pressure to gradually increase, which more closely resembles the actual production environment in oil fields. At this point, the hydrolysis degree significantly increases, and at 160 °C, it is nearly fully reacted after 6 h. The series of experiments in this study indicates that the chloroformate reacts slowly with water below 60 °C. Under high temperature and high pressure, it exhibits slow-release properties and can completely react with water. This preliminarily verifies the feasibility of using the chloroformate as an acid-generating solid agent. In the study, this self-developed laboratory method was used to determine the organic chlorine content in the hydrolysis residue of the chloroformate. The hydrolysis residue was obtained from the reaction of 0.297 g of chloroformate with 6 g of water at 160 °C for 24 h, under which conditions the chloroformate can be considered completely hydrolyzed. The results of three repeated measurements showed no presence of organic chlorine components, proving that the hydrolysis products do not contain significant amounts of organic chlorine. This preliminary verification supports its feasibility for use in oil field production. Further methods should be employed to determine the trace organic chlorine content in the hydrolysis residue, ensuring stringent control over the organic chlorine content.

### 3.2. Analysis of Free Radical Polymerization and Encapsulation of Acrylates

**Figure 3:**
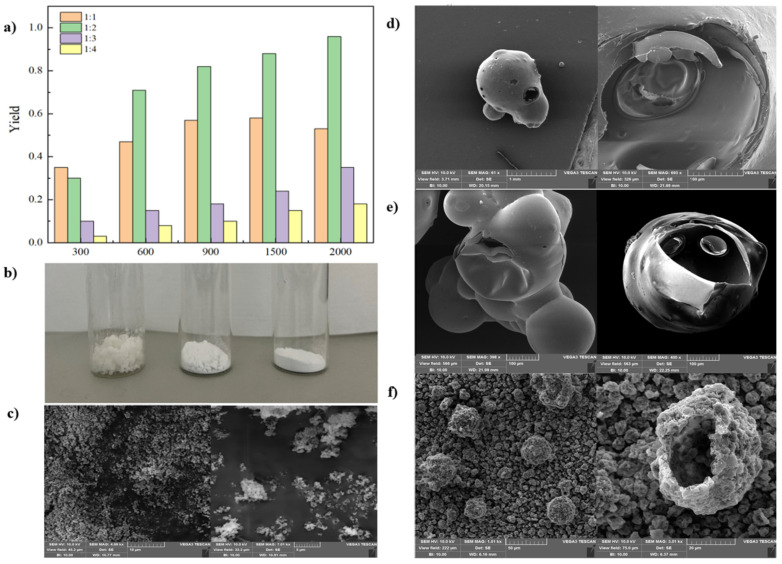
(**a**) The yield of acrylate free radical encapsulation polymerization after a 2-h reaction at 70 °C under different conditions (with polymer monomer to oil phase ratios ranging from 1:1 to 1:4). (**b**) The morphology of microcapsule products with different oil phase core materials under the same conditions (2000 rpm, polymerization at 70 °C for 2 h), **Left**: Core material is xylene. **Center**: Core material is paraffin. **Right**: Core material is bitumen. (**c**) The morphology of microcapsules prepared using a water-phase initiator for acrylate polymerization encapsulation at 300 rpm. The morphology of microcapsules with bitumen core material encapsulated at (**d**) 300 rpm, (**e**) 900 rpm, (**f**) 2000 rpm. **Left**: Overall morphology of the microcapsules. **Right**: Fractured cross-sectional morphology of the microcapsules.

First, we employed a typical suspension polymerization method, where acrylate monomers and crosslinking agents in the oil phase began to polymerize under the action of the initiator and deposit on the surface layer of the oil phase, subsequently solidifying into microcapsules that encapsulate the oil phase. We initially adopted the oil-phase initiator azobisisobutyronitrile (AIBN) for experiments. The experimental results indicated that when the rotational speed was below 1000 rpm, the produced products were uneven, irregularly shaped, large particles with high hardness and low toughness, and the encapsulation rate was low. As the rotary speed increased, the products became finer, with the particle size gradually decreasing until they appeared as uniform powder-like particles. At a rotational speed of 2000 rpm, the obtained microcapsules were uniform in size and non-sticky, with a higher yield compared to other rotational speeds. Additionally, we conducted experiments by altering the ratio of the oil phase to the polymer monomer and crosslinking agent, as well as the polymerization time, at a fixed rotational speed. When the oil phase/monomer ratio was too high, the polymerized products did not form well, and solid particulate products could not be obtained. Conversely, when the ratio was too low, the products showed significant agglomeration and contained a large amount of polyacrylate powder, inconvenient for processing and post-treatment. The yield results (calculated based on the mass of the oil phase and monomers) under different oil phase/monomer ratios and stirring speeds are shown in Figure 3a. The experiments revealed that increasing the rotational speed can improve the encapsulation yield. Simultaneously, when the proportion of the monomer is too high (with a monomer to oil phase ratio of 1:1), excessive polymerization of acrylates results in agglomeration, making it difficult to encapsulate the oil phase, thus leading to lower yield. Conversely, when the monomer proportion is too low, it is insufficient to adequately encapsulate the oil phase, also resulting in lower yields. Studies on varying the polymerization time showed that a shorter polymerization time fails to produce an adequate amount of solid material, whereas when the polymerization time exceeds 2 h, severe agglomeration of the product was observed, which is speculated to be due to over crosslinking of the polymer causing the adherence of microcapsules. Therefore, based on the multiple experiments, the optimal conditions for acrylate free radical polymerization encapsulation were determined as follows: a stirring speed of 2000 rpm, a polymer to crosslinker ratio of 9:1, a monomer to oil phase ratio of 1:2, a reaction at 70 °C for 2 h followed by curing at 85 °C for 1 h. Under these conditions, polyacrylate microcapsules with well-defined morphology can be reproducibly prepared. For comparison, ammonium persulfate was also utilized as a water-phase initiator for the encapsulation. It is important to note that when using an oil-phase initiator, the encapsulation process belongs to suspension polymerization, resulting in products with larger particle sizes. In contrast, when using a water-phase initiator, the polymerization occurs primarily at the interface between the water and oil phases and within the oil phase monomers, classifying it as dispersion polymerization. The product particle size in this case is expected to fall between that of suspension polymerization and emulsion polymerization. The experiments revealed that the method of dispersion polymerization encapsulation is less influenced by factors such as rotational speed, oil phase type, and monomer ratio. However, excessively high rotational speeds are not preferred. The encapsulated products obtained were fine powders with uniform size, exhibiting a good apparent encapsulation effect.

After determining the optimal conditions for free radical polymerization, three different oil phases—xylene, paraffin, and bitumen—were used as cores for encapsulation, with the results shown in Figure 3b. These three oil phases have high solubility for the chloroformate and theoretically can serve as suitable solvent carriers. Using the same experimental conditions for microcapsule preparation, the results indicated that xylene could not be successfully encapsulated, leading to severe agglomeration and significant xylene evaporation. Both paraffin and bitumen were successfully encapsulated. However, microcapsules with paraffin cores showed some residual leakage, resulting in a cloudy washing solution and an oily appearance after drying. In contrast, bitumen core microcapsules did not show noticeable leakage. After drying, they appeared as fine powders, indicating relatively complete encapsulation. In summary, both paraffin and bitumen can be used as solvent carriers for the encapsulation of the chloroformate, with bitumen providing the best encapsulation effect. Xylene, however, cannot serve as a core material for encapsulation, which is likely due to its higher surface tension compared to alkane-based oil phases, preventing the formation of uniform and stable microspheres in water, and thus the polymer formed during the reaction cannot cover its surface to form microcapsules. Additionally, the significant swelling of polyacrylate by xylene prevents the polymer shell from forming a stable morphology. Therefore, subsequent experiments utilized paraffin and bitumen as core materials.

To further investigate the morphology and encapsulation of the prepared acrylate microcapsules, various samples were characterized using SEM. Representative results are shown in Figure 3d–f. SEM images reveal that the samples prepared with an oil-phase initiator at low rotational speeds exhibit larger and irregular ellipsoidal particles with more surface pores and fractures. As the stirring speed increases, the microcapsule particle size significantly decreases, and the morphology becomes more spherical with less adhesion. The samples prepared under the previously determined optimal conditions for encapsulation of the chloroformate are spherical with relatively uniform size. The clear internal and external structures in cross-sections indicate good encapsulation. It is noteworthy that, compared to traditional polyacrylate microcapsules, the microcapsules prepared in this study exhibit more surface wrinkles. This is hypothesized to be due to the absence of surface morphology modifiers (such as sodium chloride) in the experiments. Since the surface wrinkles do not significantly affect the encapsulation efficiency or the high-temperature strength, no additional additives were used in subsequent encapsulation preparation processes.

In the study, we also characterized the samples prepared using a water-phase initiator through SEM. Unlike the oil-phase initiator samples, the microcapsules formed by the water-phase initiator are of well-defined morphology, much smaller in diameter, approximately 0.5 μm, as shown in Figure 3c. These findings suggest that both water-phase and oil-phase initiators can be adopted to successfully prepare microcapsules. Depending on the desired particle size, different initiators can be selected for preparation.

**Figure 4:**
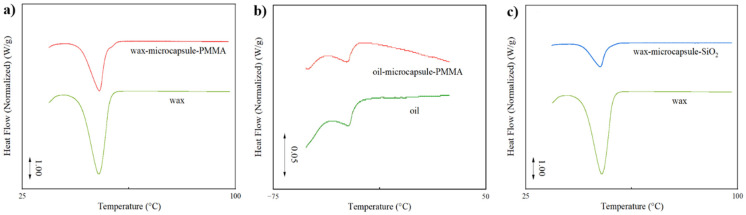
Differential scanning calorimetry (DSC) test results of microcapsules with (**a**) paraffin core material and pure paraffin, (**b**) bitumen core material and pure bitumen. (**c**) DSC test results of silica microcapsules with paraffin core material and pure paraffin.

In addition, differential scanning calorimetry (DSC) was used to measure the encapsulation efficiency of the different microencapsulation methods on the oil phase. Bulk microcapsule samples with paraffin or bitumen as core, prepared using oil-phase initiators under the optimal conditions mentioned above, were analyzed. The test results are shown in Figure 4a, b. During testing, the temperature was increased from below the melting point of the oil-phase core material (approximately 50 °C for paraffin and −60 °C for bitumen). The heat flow curves were integrated to obtain the melting enthalpy of the samples (unit: J/g), and the encapsulation efficiency of the oil phase in the microcapsule samples was calculated according to Equation (1) (defined as the ratio of the mass of the oil phase to the total mass of the microcapsules). The experimental results indicate that an encapsulation efficiency of around 60% was achieved using acrylate free radical polymerization for both paraffin and bitumen core materials, indicating a relatively high degree of encapsulation. In summary, the method of using acrylate free radical polymerization for encapsulating oil phase droplets has proven to be generally successful.

**Figure 5:**
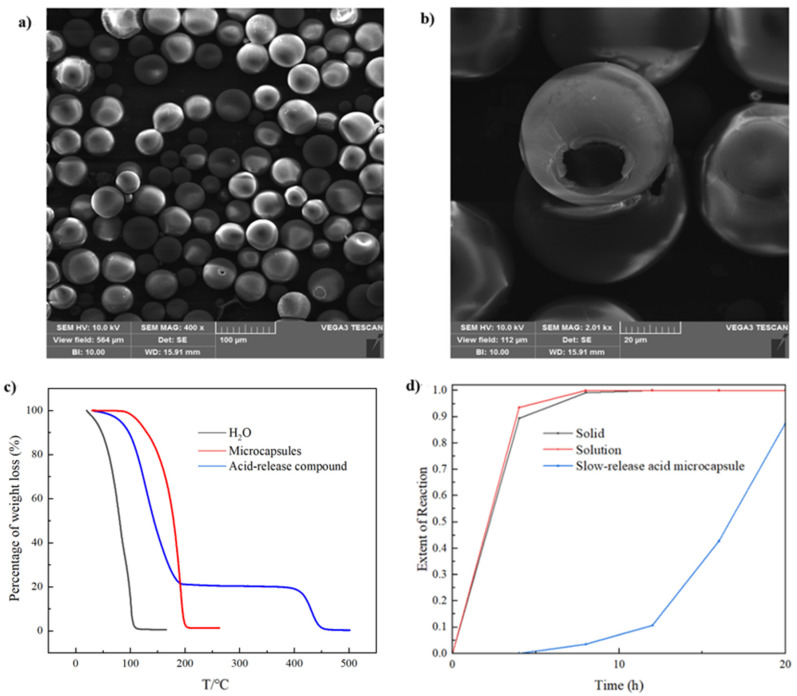
(**a**,**b**) The morphology of microcapsules prepared by the phase separation encapsulation method. (**c**) DTA scanning images of water, bulk chloroformate, and microcapsule samples prepared by the phase separation method. (**d**) Hydrolysis degree of different forms of chloroformate and encapsulated acid-release system at 70 °C over time.

Based on the aforementioned experiments, the oil phase solution of the chloroformate was used instead of pure oil phase for encapsulation, resulting in microcapsules similar to those obtained in previous experiments. However, compared to the products obtained from pure oil phase as core, the yield of the microcapsules encapsulating the chloroformate significantly decreased. The reason is that considerable hydrolysis of the chloroformate occurred at the polymerization temperature, leading to a reduced encapsulation rate. Samples were subjected to hydrolysis at 70 °C for 20 h, with the pH of the residual liquid measured every 4 h. The results were compared with the hydrolysis results of the chloroformate itself, as shown in Figure 5d. The prepared encapsulated slow-release acid system did not produce acid after 5 h of hydrothermal treatment at 70 °C. The delayed acid generation capability was significantly stronger than that of the chloroformate itself, meeting the requirements for slow and complete acid release at higher temperatures. This satisfies the industrial requirements of slow-release acid: No acid generation at low temperatures and slow acid release at high temperatures.

### 3.3. Analysis of Silica Precipitation Polymerization and Encapsulation

Due to the high temperatures and complex procedures associated with the acrylate encapsulation method, this study also explores a milder, simpler inorganic encapsulation method. This method generates cross-linked silica as the shell layer through the in-situ formation and subsequent dehydration of silicic acid. There have been numerous studies on silica encapsulation microcapsules, commonly using the sol-gel method [17,18]. This involves the in-situ hydrolysis of silicon-containing materials like tetraethyl orthosilicate to generate silica gel for encapsulation. However, due to the high hydrolytic reactivity of silicate species and their tendency to react with the chloroformate in a basic environment [19], they are unsuitable for encapsulation. Therefore, this study employs the less commonly used precipitation method for encapsulating the chloroformate, which involves the in-situ generation of silicic acid by adding sodium silicate solution dropwise to an acidic solution. Through electrostatic interactions with surfactants adsorbed on the surface of the oil phase droplets, the silicic acid precipitates onto the surface of the oil phase droplets, forming silica shell microcapsules upon dehydration.

The reaction time and temperature required for the silica precipitation method are significantly lower than those needed for the acrylate free radical polymerization encapsulation method. During the experiments, various conditions, such as stirring speed and oil phase composition, were altered, and multiple preparation experiments were conducted. It was found that the silica encapsulation capability was relatively poor. Notably, when xylene was used as the core material, significant silica agglomeration occurred, resulting in no encapsulation. Using alkane core materials produced relatively fine textures, with bitumen showing the best results. Additionally, the experiments revealed that at low stirring speeds, the resulting product was a viscous mass, while at high stirring speeds, it was a finer dry powder. This indicates that the silica encapsulation capability varies with stirring speed.

Similar to the acrylate encapsulation system, DSC was used to determine the encapsulation efficiency of the well-formed silica precipitation-encapsulated microcapsules for the oil phase. Due to the relatively low encapsulation strength and efficiency of the silica precipitation method and the minimal melting enthalpy of bitumen, meaningful data could not be obtained using bitumen as the oil phase. Therefore, in the experiments, only paraffin was used as the core material to obtain the encapsulation efficiency of the silica microcapsules, as shown in Figure 4c. The DSC test results indicated that the encapsulation efficiency of silica microcapsules with paraffin as the core material was only about 30%, reflecting a low encapsulation efficiency.

### 3.4. Phase Separation Encapsulation

The phase separation encapsulation method is a novel technique for preparing microcapsules. This method involves dissolving both the core and shell materials in an oil phase, which is then emulsified in a large volume of water phase. At a relatively low temperature, the oil solvent evaporates, causing the shell material to precipitate and encapsulate the core material under the influence of surface tension, thereby forming microcapsules with controllable wall thickness and diameter [20]. The detailed principles of the phase separation method have been explained in other literature and will not be elaborated here [21,22]. The key features of this encapsulation method include the ability to prepare microcapsules without chemical reactions, a relatively clean reaction process without harmful by-products, and mild conditions with low energy consumption [23]. In this study, we successfully employed this method to prepare microcapsules with the chloroformate as the core material. This method presents a feasible option for the low-cost production of slow-release acid materials.

In the experiments, polystyrene was used as the shell material and dichloromethane as the oil phase solvent. The specific experimental procedures and parameters are detailed in the Methods section. This method consistently produced well-formed microcapsules with smooth surfaces and diameters uniformly distributed between 30–50 μm and wall thicknesses of approximately 1–2 μm, as observed under SEM, as shown in the thermo-gravimetric analysis (TGA) of the prepared samples, which revealed a weight loss of about 70% at the complete decomposition temperature of chloroformate in Figure 5c. Due to the preparation process, the samples contained a small amount of moisture, and the encapsulation efficiency of the chloroformate in the samples was estimated to be around 50–60%, indicating a substantial encapsulation amount. In summary, the slow-release acid microcapsules prepared by the phase separation method exhibit excellent morphology, a simple preparation process, and a high encapsulation efficiency of the core material. Further research is warranted to explore this method’s potential.

## 4. Conclusions

In this study, we investigated the feasibility of the application of a slow-release acid system prepared with a novel slow acid-generating material as the core in detail. We first investigated the hydrolysis and acid generation performance of the acid-release material, mainly chloroformate, under different conditions. Later, by encapsulating the chloroformate to prepare microcapsules, we successfully developed a novel slow-release acid system that does not generate acid at room temperature but slowly produces acid at high temperatures. Through experiments, we verified the feasibility of using the chloroformate as an acid-generating material at first. The hydrolysis of the bulk chloroformate is relatively slow at room temperature, but the hydrolysis rate significantly increases at rising temperature, and complete hydrolysis occurs after 6 h at temperatures exceeding 160 °C. Low pH values can inhibit the hydrolysis of the chloroformate to some extent; therefore, when choosing an encapsulation method, we preferred low-temperature rapid encapsulation under acidic conditions to reduce the reaction between the chloroformate and water. Additionally, we developed a chemical oxidation method to measure the organic chlorine content in the hydrolysis residue of the chloroformate. This method can roughly characterize the content of organic chlorine. The preliminary characterization results indicate that the hydrolysis residue of the chloroformate does not contain significant amounts of organic chlorine. Subsequently, we coated chloroformate in a variety of ways to obtain microcapsules with good sustained-release performance. Using the acrylate free radical polymerization method to encapsulate different oil phases, we found that when using an oil-phase initiator, fine microcapsules encapsulating paraffin or bitumen could be prepared under conditions of a 2000 rpm stirring speed, a monomer-to-oil ratio of 1:2, and at a reaction temperature of 70 °C for 2 h. The particle size was about 0.1 mm, and the encapsulation efficiency was about 60%. The results indicate that different conditions and initiators can be used to prepare the encapsulated microcapsules of different particle sizes according to the requirements. Using the acrylate free radical polymerization method to encapsulate the acid-release mixture, we prepared microcapsules with fine morphology and stable properties, with an encapsulation content of about 20%. Compared to the acrylate polymerization encapsulation method, the silica precipitation polymerization method showed an encapsulation efficiency of only 30%, indicating poor encapsulation efficiency. Furthermore, the phase separation method was applied to prepare microcapsules with fine morphology and diameters ranging from 30 to 50 μm. The TGA results indicated a high encapsulation efficiency of 50–60%, which deserves further research. The results of a series of studies on the prepared slow-release acid system show that the microcapsules with polystyrene as the shell could effectively coat the new acid-generating mixture, and the coating efficiency could reach more than 60%. We found that the slow-release acid-generating performance was excellent, and the continuous acid-generating capacity at 70 °C could exceed 20 h. Therefore, in contrast to traditional slow-release acids, ordinary acid solutions, and uncoated chloroformates, the solid microcapsule system we prepared has satisfying slow acid release performance, while the production process is simple and the storage and use are very convenient. At high temperatures, this system will completely hydrolyze to produce small molecular products, which will not have any adverse effects on the environment.

In summary, this study developed a novel slow-release acid system for oilfield acidizing and fracturing. Compared to traditional slow-release acid systems, the newly prepared slow-release acid system generates acid more slowly at high temperatures, produces cleaner products, and is more convenient to use, offering significant application advantages. Future research should further optimize and refine the preparation process to improve the encapsulation efficiency and enhance the slow-release performance of the prepared acid system. As a high-performance, controllable acid-generating, and environmentally friendly oilfield additive, it is expected to play a significant role in unconventional oil and gas extraction and oilfield modification applications.

## Data Availability

The original contributions presented in this study are included in the article. Further inquiries can be directed to the corresponding author.

## References

[1] Zhao J. (2012). Conception, classification and resource potential of unconventional hydro carbons. Nat. Gas Geosci..

[2] Yang X., Zhou M. (2012). A new acid fracturing technique for carbonate reservoirs with high-temperature and deep layer. Acta Pet. Sin..

[3] Buijse M.A., van Domelen M.S. Novel Application of Emulsified Acids to Matrix Stimulation of Heterogeneous Formations. Proceedings of the SPE Formation Damage Control Conference.

[4] Ahmed M., Sultan A., Qiu X., Sidaoui Z., Al-Amri A.A. A Novel Emulsified Acid for Deep Wells Stimulation: Rheology, Stability, and Core flood Study. Proceedings of the SPE Kingdom of Saudi Arabia Annual Technical Symposium and Exhibition.

[5] Yuan L., Wang Y., Li Q. (2019). Evaluation of a control-released in-situ generated acid tablet for acid fracturing. J. Pet. Sci. Eng..

[6] Rabie A.I., Saber M.R., El-Din H.A.N. A new environmentally friendly acidizing fluid for HP/HT matrix acidizing treatments with enhanced product solubility. Proceedings of the SPE International Symposium on Oilfield Chemistry.

[7] Quraishi M.A., Shukla S.K. (2009). Poly (aniline-formaldehyde): A new and effective corrosion inhibitor for mild steel in hydrochloric acid. Mater. Chem. Phys..

[8] Wang S., Zhang D., Guo J., Guan B. (2018). Experiment and analysis of the reaction kinetics of temperature control viscosity acids with limestone. J. Pet. Sci. Eng..

[9] Gonzalez M.E., Looney M.D. (2001). Use of Encapsulated Acid in Acid Fracturing Treatments. U.S. Patent.

[10] Ammala A. (2013). Biodegradable Polymers as Encapsulation Materials for Cosmetics and Personal Care Markets. Int. J. Cosmet. Sci..

[11] Johnson L.M., Shepherd S.D., Rothrock G.D. (2016). Core/shell systems for delayed delivery of concentrated mineral acid. SPE Prod. Oper..

[12] Shafiq M.U., Chong Y.J., Mahmud H.K.B. (2019). Application of emulsified acids on sandstone formation at elevated temperature conditions: An experimental study. J. Pet. Explor. Prod. Technol..

[13] Shafiq M.U., Mahmud H.B. (2017). Sandstone matrix acidizing knowledge and future development. J. Pet. Explor. Prod. Technol..

[14] Sultan A.S., Sidaoui Z. (2019). Emulsified Acid Comprising Waste Oil for Acidizing Geological Formations. U.S. Patent.

[15] Sabhapondit A., Guillen J.R., Prakash C. Laboratory optimization of an emulsified acid blend for stimulation of high-temperature carbonate reservoirs. Proceedings of the North Africa Technical Conference and Exhibition.

[16] Sokhanvarian K., Pummarapanthu T., Arslan E., Nasr-El-Din H.A., Shimek N., Smith K. A new in-situ generated acid system for carbonate dissolution in sandstone and carbonate reservoirs. Proceedings of the International Conference on Oilfield Chemistry.

[17] Belessiotis G.V., Papadokostaki K.G., Favvas E.P., Efthimiadou E.K., Karellas S. (2018). Preparation and investigation of distinct and shape stable paraffin/SiO2 composite PCM nanospheres. Energy Convers. Manag..

[18] Fang Y.T., Kuang S.Y., Zhang Z.G., Gao X.N. (2007). Preparation of nano-encapsulated phase change materials. Chem. Ind. Eng..

[19] Sharma A., Tyagi V.V., Chen C.R., Buddhi D. (2009). Review on thermal energy storage with phase change materials and applications. Renew. Sustain. Energy Rev..

[20] Wang Y., Guo B., Wan X., Xu J., Zhang Y. (2009). Janus-like polymer particles prepared via internal phase separation from emulsified polymer/oil droplets. Polymer.

[21] Teshima K. (2003). Unique Microcapsule Toner Synthesized by Liquid Phase Separation. Adv. Eng. Mater..

[22] Atkin R., Davies P., Hardy J., Vincent B. (2004). Preparation of Aqueous Core/Polymer Shell Microcapsules by Internal Phase Separation. Macromolecules.

[23] Wang Y., Zhao P., Zhang S., Zhu K., Shangguan X., Liu L., Zhang S. (2022). Application of Janus Particles in Point-of-Care Testing. Biosensors.

